# Multisession Anodal tDCS on the Right Temporo-Parietal Junction Improves Mentalizing Processes in Adults with Autistic Traits

**DOI:** 10.3390/brainsci12010030

**Published:** 2021-12-28

**Authors:** Iván Padrón, Enrique García-Marco, Iván Moreno, Agustina Birba, Valentina Silvestri, Inmaculada León, Carlos Álvarez, Joana López, Manuel de Vega

**Affiliations:** 1 Instituto Universitario de Neurociencia, Universidad de La Laguna, 38200 La Laguna, Spain; ivpadron@ull.edu.es (I.P.); egarciam.psicologia@gmail.com (E.G.-M.); ivanzevenzui@hotmail.com (I.M.); ileons@ull.es (I.L.); calvarez@ull.es (C.Á.); joana.lopez.p91@gmail.com (J.L.); 2 Facultad de Ciencias de la Salud, Universidad Europea de Canarias, 38300 La Orotava, Spain; 3 Facultad de Psicología, Universidad Nacional de Educación a Distancia (UNED), 28040 Madrid, Spain; 4 Cognitive Neuroscience Center (CNC), Universidad de San Andrés, Buenos Aires B1644BID, Argentina; agustina.birba@gmail.com; 5 National Scientific and Technical Research Council (CONICET), Buenos Aires C1033AAJ, Argentina; 6 Department of Psychology, University of Milan-Bicocca, Piazza dell’Ateneo Nuovo 1, 20126 Milan, Italy; v.silvestri11@campus.unimib.it; 7 Department of Psychology, Faculty of Health Sciences, University of Hull, Hull HU6 7RX, UK

**Keywords:** autism spectrum disorder, brain stimulation, tDCS, mentalizing skills, theory of mind, false belief, self-other judgments

## Abstract

Persons with autism spectrum disorder (ASD) have impaired mentalizing skills. In this study, a group of persons with ASD traits (high-AQ scores) initially received sham tDCS before completing a pre-test in two mentalizing tasks: false belief and self-other judgments. Over the next week, on four consecutive days, they received sessions of anodal electrical stimulation (a-tDCS) over the right temporo-parietal junction (rTPJ), a region frequently associated with the theory of mind. On the last day, after the stimulation session, they completed a new set of mentalizing tasks. A control group (with low-AQ scores) matched in age, education and intelligence received just sham stimulation and completed the same pre-test and post-test. The results showed that the high-AQ group improved their performance (faster responses), after a-tDCS, in the false belief and in the self-other judgments of mental features, whereas they did not change performance in the false photographs or the self-other judgments of physical features. These selective improvements cannot be attributed to increased familiarity with the tasks, because the performance of the low-AQ control group remained stable about one week later. Therefore, our study provides initial proof that tDCS could be used to improve mentalizing skills in persons with ASD traits.

## 1. Introduction

The human brain has developed extraordinary mentalistic capacities, far superior to those of other species of primates, to deal with our complex social world. The so-called “theory of mind” (herein ToM) is a cognitive skill that allows us to navigate the social world while continuously tracking our own and others’ mental states and engaging in social reasoning [1,2]. ToM implies mental perspective-taking, that is, the ability to dissociate one’s own perceptions, beliefs and intentions from those of others. However, persons with autism spectrum disorders (ASD) have a chronic impairment in their ToM processes. Specifically, they are less capable of attributing mental states to others or themselves [3]. In this study, we tested whether the application of multisession non-invasive brain stimulation in the right temporoparietal junction, a specific brain region of the ToM network, could improve performance in mentalizing tasks in persons with high scores in ASD. 

### 1.1. The Neural Bases of ToM

We can distinguish two main neural networks involved in mentalizing processes: the affective ToM, which is mainly responsible for emotional empathy, and the cognitive ToM, which is assumed to compute others’ mental states, dissociating them from ours and enabling our complex social reasoning capabilities [4,5,6]. Especially relevant to the current research is the cognitive ToM, selectively recruited to distinguish self-reference representations and other-reference representations, and to reason about others’ mental states (beliefs, intentions) and their consequences. The cognitive ToM comprises several broadly distributed brain regions, especially in the right and medial cortex, namely, the right temporo-parietal junction (rTPJ), the posterior right superior temporal sulcus (STS), the right extrastriate body area (EBA), the right medial prefrontal cortex (rMPFC) and the precuneus (PC) [7]. According to many researchers, the rTPJ plays a privileged role in the social brain, being responsible for understanding the beliefs and intentions of others, managing the representation of the self and the other, monitoring the other’s visual perspective, as well as empathic perspective (see meta-analysis by [8]). For instance, neuroimaging studies have reported that false belief tasks, compared to false photographs, induce activations in the rTPJ and, to a lesser extent, the left TPJ [4,9,10,11,12,13,14,15,16]. In the same vein, there is evidence that the right TPJ and to some extent the left TPJ [17] is actively involved in tasks demanding self-other distinctions (see review by [18,19]).

Since autism is characterized by impaired cognitive ToM, some studies have explored the brain functional organization of persons with ASD, compared to control persons, while dealing with ToM tasks. For instance, neuroimaging studies by [20,21] asked ASD persons and controls to make physical and mentalizing judgments that referred to the self or referred to the other (e.g., the British Queen). Control participants showed a distinctive pattern of activations of medial structures, such as the ventromedial prefrontal cortex (vmPFC) and the mid-cingulate cortex (mCC), for self-reference compared to other-reference judgments, and in the rTPJ for mentalizing compared to physical judgments. By contrast, ASD participants recruited the vmPFC equally for self and other judgments, and the rTPJ for mentalizing and physical judgments. However, other neuroimaging studies did not find systematic differences between control and ASD persons in brain activations during the performance of ToM tasks (e.g., [22]). Although these findings are not entirely conclusive, they suggest that the functional organization of the neural circuits of ToM may involve atypical features in ASD persons, such as less functional specificity, compared to control persons, which could be partially responsible for their impaired social cognition. Yet, the rTPJ appears to be involved when ASD participants perform ToM tasks, which justifies our choice of this region as a target for multisession tDCS. 

### 1.2. Non-Invasive Brain Stimulation of ToM in ASD Persons

The study of the efficiency of non-invasive brain stimulation to improve social cognition both in control individuals and in persons with ASD is a growing field. According to a recent review [23], some studies report promising improvements in persons with ASD in visual, motor and affective processes as a consequence of repetitive transcranial magnetic stimulation (rTMS). For example, [24] applied excitatory rTMS to persons diagnosed with ASD for two weeks of daily sessions over the medial prefrontal cortex (a part of the mentalizing network, structurally and functionally connected to the TPJ), showing potential benefits, as suggested by the self-reported reduction of social related symptoms and self-oriented anxiety. 

An alternative technique is transcranial direct current stimulation (tDCS). Although tDCS has the significant drawback of offering much less focal stimulation than rTMS, it also has some advantages, such as having relatively long-lasting effects—about an hour after 12 min of stimulation [25]—a generally noticeable impact on performance and the possibility to use excitatory (anodal) or inhibitory (cathodal) stimulation [26,27]. In the social domain, the use of tDCS remains relatively limited, and to date, it has been mainly aimed at experimentally inducing changes in cognitive and social behavior in neurotypical individuals, to understand better the neural mechanisms of ToM. For example, anodal tDCS on the rTPJ improves interpersonal perspective taking [28], facial emotion processing [29], and detecting the mismatch between self-related and other-related representations [30]. Cathodal tDCS over the same region impairs performance in ToM and cognitive empathy tasks [31]. Most relevant to the current research was a recent study that applied high-definition tDCS over the rTPJ to persons whose Autism Quotient (AQ) scores had been previously measured [32]. The authors observed that the stimulation-induced changes in performance in ToM tasks and in EEG signatures correlated with the AQ scores. Namely, high-AQ participants showed poorer performance on ToM tasks before the stimulation, and cathodal tDCS further affected their performance in the attribution of mental states. On the other hand, anodal tDCS applied on the left TPJ regulated the delta rhythms of EEG [33], also correlating with AQ scores, during an emotion recognition task. Therefore, it seems that stimulation to TPJ interacts with attentional processes and social cognition, aspects that are especially relevant in persons with autism.

### 1.3. The Present Study

As we have seen, evidence of the therapeutic efficacy of non-invasive brain stimulation to improve social cognition in persons with autistic traits is still scarce. In this study, we developed for the first time a multisession intervention with anodal tDCS over the right TPJ in persons with high-AQ scores. We measured their performance in validated mentalizing tasks before and after the treatment. A group of low-AQ persons served as controls, receiving sham tDCS and performing the same mentalizing tasks in two sessions with the same time delay as the high-AQ group. 

For this study, we selected two well-known tasks that are representative of cognitive ToM skills, that is, the false belief task and the self-other judgment task, which are associated with activations in the rTPJ. Both tasks involve a dissociation between representations of the self and representations of the other. The false belief paradigm consists of presenting stories in which a protagonist performs an action based on a false belief because he/she is unaware of a critical event, which the reader/listener knows. For example, “Mary puts her chocolate in the top drawer of the table, and while she was at the school, her mother moved it to the bottom drawer of the table. When Mary comes back home, will she look for the chocolate in the top drawer?” To correctly answer this question, participants need to recreate the outdated belief of the protagonist to understand her actions (looking in the top drawer) even though they know the chocolate is actually in the bottom drawer. These stories are contrasted with stories of “false photos” that also require the representation of outdated content but do not involve monitoring the characters’ beliefs. For example, “An old photo showed a house with a front door and two windows on the sides. In 2010 one of the windows was replaced with a new door. Did the old photo show the two doors?” Therefore, false belief stories and false photographs are similar in their general difficulty, logical complexity and inhibitory demands, but differ in the need to think about someone’s thoughts (false beliefs) [9,22,34]. We expect that the anodal tDCS on rTPJ can improve the performance of the high-AQ participants in the false belief task, but not in the false photo task.

The second task involves self-reference or other-reference judgments of physical or mental features. The participants were asked to judge to what extent a physical characteristic (e.g., having blue eyes) or a mental characteristic (e.g., having a strong will) applies to you (self-reference) or to a famous person (other-reference). This task requires that the participant know the other’s mental states (beliefs, intentions, emotions and mental features), which frequently differ from our own [35]. We predict that the anodal tDCS improves the performance of the high-AQ participants in the self-other judgments referring to mental features, but not in those referring to physical features, which do not require ToM processes.

As for the low-AQ group, it serves as a passive control (with participants receiving only placebo stimulation) to assess whether there is any improvement between the initial and the final test of the mentalizing tasks, as a simple consequence of familiarization or learning. The expectation is that their performance would remain stable in ToM tasks. 

## 2. Materials and Methods

### 2.1. Participants

Forty-two Spanish-speaking participants, 21 with high-AQ scores and 21 with low-AQ scores serving as the control group, were selected. All were right-handed, had normal or corrected-to-normal visual acuity and had signed an informed consent form. Participants were screened via safety guidelines for transcranial stimulation [36] and the MINI International Neuropsychological Interview [37]. About 2000 university students completed the Autism Spectrum Quotient or AQ [38], through an online platform, and those with a score higher than 32 were selected as the high-AQ. participants. We initially found only 12 participants, so we changed the criterion to a score of at least 30, which allowed us to incorporate nine more participants. The mean score of the resulting high-AQ group was 33.09, SD = 2.99, range 30–38. As for the control group, the mean score of the 2000 participants was calculated and a cutoff point of 2 SDs below the mean was established, resulting in 21 participants with a mean score of 17.66, SD = 1.46, range 16–19. Once selected, the participants went to the laboratory to complete the Empathy Quotient or EQ [39] and the Raven general intelligence test [40] and sign the informed consent document. As could be expected, the two groups statistically differed in AQ scores (F (1, 40) = 449.384; *p* = 0.0001), and in the EQ scores (high-AQ: M = 43.52, SD = 10.92; low-AQ: M = 29.09, SD = 12.19; F (1, 40) = 16.225; *p* = 0.0001). However, they did not differ in age (high-AQ: M = 22.47, SD = 4.58; low-AQ: M = 20.47, SD = 2.18; F (1, 40) = 3.253; *p* = 0.079), or Raven scores (high-AQ: M = 100.7, SD = 7.96; low-AQ: M = 98.57, SD = 6.88; F (1, 40) = 0.870; *p* = 0.357). 

### 2.2. Stimulation Protocol

**Figure 1:**
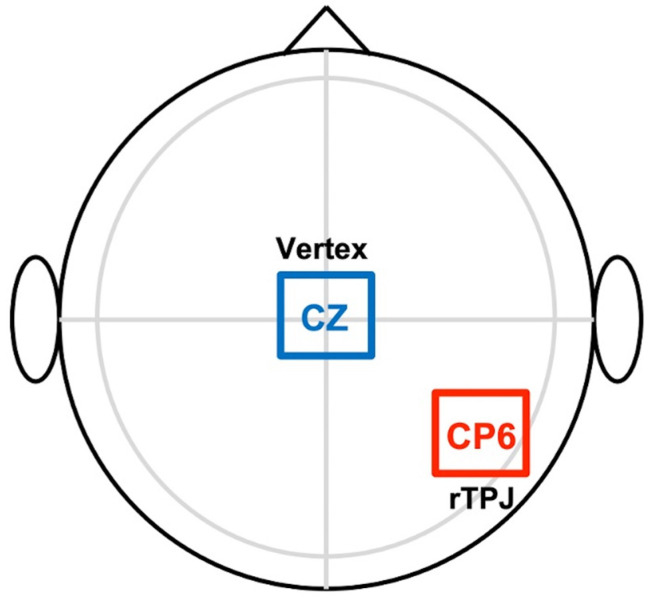
Montage of tDCS electrodes, for both the sham and the active condition.

The active stimulation sessions consisted of 20 min of anodal tDCS over the right TPJ, with the cathode electrode placed at the vertex, that is, at the CP6 and Cz positions in the 10/20 international localization system, respectively (Figure 1). The electrode size was 25 cm^2^ and the current intensity was 2 mA, which involves a current density of 0.08 mA/cm^2^. The sham sessions had identical electrode setup and timing, except that real stimulation only took place during the first 30 s. Afterward, participants completed a survey regarding the tolerability of stimulation and guessing the stimulation condition. None of the participants reported special discomfort, and all believed they had been stimulated.

**Figure 2:**
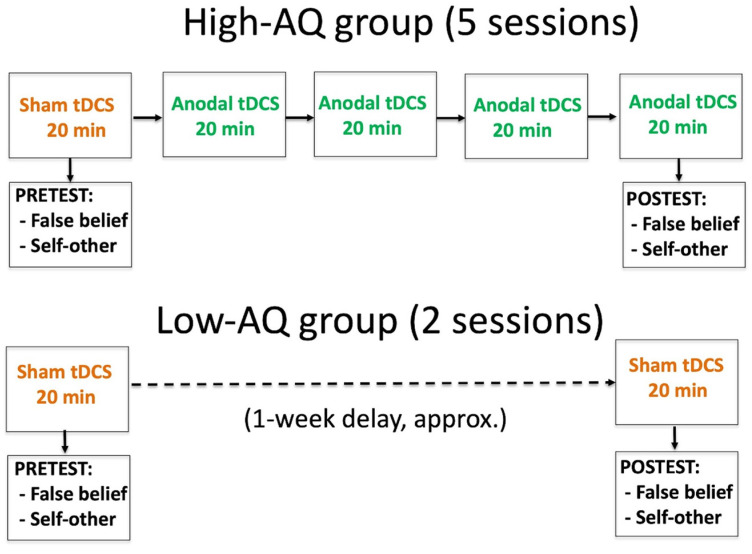
Experiment timeline for the high-AQ and low-AQ groups.

Figure 2 shows the experiment timeline for the two groups of participants. On the first day, both the high-AQ group and the low-AQ group received 20 min of sham stimulation, followed by the pre-test of the mentalizing tasks (false belief, self-other judgments). The next week, the high-AQ group received four 20-min sessions of anodal tDCS held over consecutive days. On the last day, the stimulation was followed by the post-test of a new set of the same mentalizing tasks. As for participants in the low-AQ group, since they were playing the role of controlling learning, they only received sham stimulation in the first and last sessions, each followed by the pre-test and post-test of the mentalizing tasks. The time elapsed between the pre-test and post-test sessions was similar in the two groups: 9 days (SD = 3.71) in the high-AQ group, and 8.42 days (SD = 3.31) in the low-AQ group (F (1, 40) = 0.277; *p* = 0.602). 

### 2.3. Materials and Design

Belief task. In this task, a repeated measures factorial design was used, with 2 Test (Pre-test vs. Post-test) × 2 Modality (False belief vs. False photo) × 2 Response polarity (Yes vs. No) as within-participant factors and 2 Group (high-AQ vs. low-AQ) as a between-participant factor. Two sets of 20 stories (A and B) were used in a counterbalanced way for the pre-test and the post-test sessions. Set A consisted of the original stories created by [9], translated to Spanish, whereas set B comprised 20 stories created for the experiment, with identical structure and experimental manipulations as the previous set. Half of the stories in each set described a false belief situation, and the remaining stories described a false photograph or map. The false belief and the false photo stories required participants to consider a dual representation of a false and a real situation. The critical difference was that the false content was a character’s false belief in the former and false information provided by a photograph in the latter. At the end of each story, a statement describing an outcome that was consistent or inconsistent with the reported situation was presented, and the participant judged whether it was correct or not (see examples in Table 1). The number of ‘yes’ and ‘no’ questions was the same for false belief and false photo stories, which were presented in random order. The participants answered the question by pressing a previously assigned key on the keyboard (S = yes, L = no) in spatial positions consistent with the corresponding words on the screen. The reaction times in milliseconds (RTs) and response accuracy (probability of correct response) were collected to be used as dependent measures.

Self-other judgment task. In this task, the design included 2 Test (Pre-test vs. Post-test) × 2 Reference (You vs. Other) × 2 Feature (Mental vs. Physical) as within-participant factors and 2 Group (high-AQ vs. low-AQ) as between-participant factor. Two sets of self-other questions were adapted from an earlier version used by [21]. Participants were asked to make mental or physical judgments about themselves or a famous person (Lady Gaga and Leo Messi, presented in a counterbalanced order in the two test sessions). At the beginning of the assigned set, they read a short biography of the famous person and were told that they would be asked to rate the likelihood that such person (the other) or the participant (you) had certain physical (stature, hair color, etc.) or mental features (opinions, likes and dislikes). For example, a ‘’you-physical’’ judgment could be ‘’How likely are you to be muscular?’’, whereas an ‘’other-mental’’ judgment could be ‘’How likely is she to enjoy the adrenaline rush of taking risks?’’. Before each trial, the label ‘’YOU’’ or the famous person’s name (“LADY GAGA” or “LEO MESSI”) appeared on the screen for 2 s (letter size 45 pt), to establish the reference for the incoming question. The RTs were collected to be used as the dependent measure. In this task, there were no accuracy measures since correctness criteria were not applicable. 

In each task set, there were 20 items in each type of question (you-mental, you-physical, other-mental, other-physical), which is a total of 80 items. Participants issued a judgment on a scale of 1–4 (1 = not likely, 4 = very likely). To encourage participants to engage in the task, they were told that their judgments would be compared with the answers given by the famous persons in various interviews, and they would receive an ‘accuracy score’ at the end of the task. This ‘’score’’ was randomly generated and displayed on the screen at the end of the task. 

## 3. Results

Global analyses of variance (ANOVAs) were performed in each task for reaction times and accuracy (only in the belief task), including Group (high-AQ and low-AQ), Test (pre-test and post-test) and the specific variables of each task: Modality (belief vs. photo) in the belief task and Feature (mental vs. physical) and Reference (you vs. other) in the self-other task. To explore the resulting interactions, separate ANOVAs were applied to each group separately, followed by pairwise comparisons (*t*-tests), to assess the effects of the test (pre-test vs. post-test) for each experimental condition in each group, and to contrast the two groups (high-AQ and low-AQ) for each experimental condition. 

### 3.1. Belief Task

The response polarity was a control variable manipulated to encourage the participants to pay full attention to the two alternative states: the real situation and the false belief/photo. Therefore, it was collapsed in the analyses of variance (ANOVAs) which focused on the variables with theoretical interest: Group × Modality × Test. However, the statistics referring to response polarity are offered in the Supplementary Materials.

**Figure 3:**
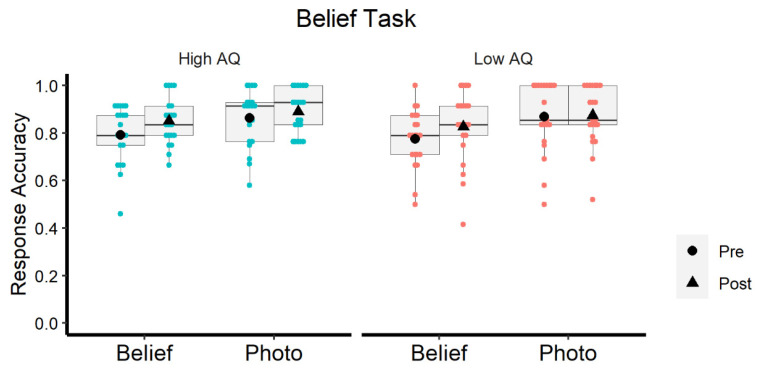
Probability of correct responses in the belief task, as a function of Group (high-AQ and low-AQ), Modality (false belief and false photo) and Test (pre- and post-test).

Concerning response accuracy, both groups showed similar high performance (high-AQ: M = 0.849; low-AQ: M = 0.837; F < 1). There was a slight improvement from the pre-test (M = 0.82, SD = 0.17) to the post-test (M = 0.86, SD = 0.16), which was statistically significant (F (1, 40) = 4.947; *p* = 0.032; η² = 0.110), suggesting certain familiarization with the task that was independent of the stimulation protocol. However, the crucial Test × Group interaction was not significant (F < 1), which means that the learning effect was similar in both high-AQ and low-AQ participants (Figure 3). Furthermore, both groups were more accurate in the photo (M = 0.87, SD = 0.15) than in the belief modality (M = 0.81, SD = 0.18); F (1, 40) = 17.236; *p* = 0.0001; η² = 0.301, which is consistent with other results in the literature [9]. The accuracy results are illustrated in Figure 3.

As for the RTs, the high-AQ group showed a nonsignificant trend to respond more slowly than the low-AQ group (M = 4113 ms and M = 3903 ms, respectively; F (1, 40) = 1.47; *p* = 0.232). Furthermore, the t-test contrasting high-AQ and low-AQ for each experimental condition substantiated a significant group difference for the false belief items in the pretest session (high-AQ: M = 4561 ms; SD = 712; low-AQ: M = 3986 ms; SD = 541; t (40) = 2.946, *p* < 0.005), while the two groups did not differ in the false photo items (high-AQ: M = 4088 ms; SD = 724; low-AQ: M = 3903 ms; SD = 540; t (40) = 0.878, *p* = 0.354). 

There were significant main effects of Test: F (1, 40) = 16.765; *p* = 0.0001; η² = 0.295 (slower responses in the pre-test than the post-test), and Modality: F (1, 40) = 6.092; *p* = 0.018; η² = 0.132 (slower responses to false beliefs than to false photos). Most important, the interaction Test × Group was also significant (F (1, 40) = 7.597; *p* = 0.009; η² = 0.160), since the difference between pre- and post-test was much larger in high-AQ (Pre: M = 4234 ms, SD = 840 ms; Post: M = 3901 ms, SD = 742 ms) than in low-AQ participants (Pre: M = 3944 ms, SD = 602 ms; Post: M = 3862 ms, SD = 859 ms). To explore this interaction in detail, separate ANOVAs were performed for the high-AQ and the low-AQ group. 

**Figure 4:**
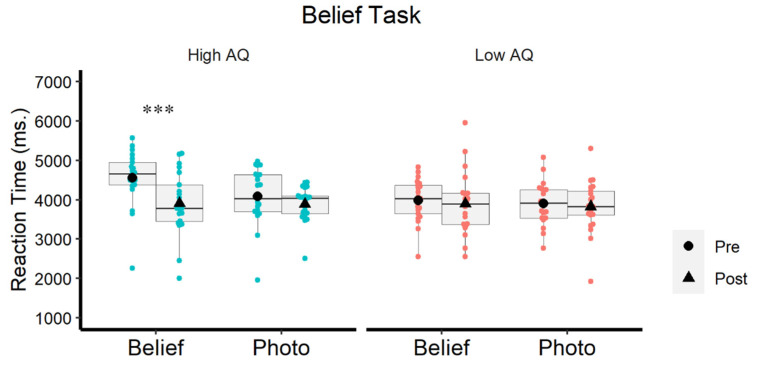
Reaction times in the belief tasks in the high-AQ group (**left**) and the low-AQ group (**right**) as a function of Modality (false belief and false photo) and Test (pre- and post-test). Only significant effects of test are signaled (*** *p* < 0.0001).

For the high-AQ group, there was a main effect of Test (F (1, 20) = 22,713; *p* = 0.005; η² = 0.532) and Modality (F (1, 20) = 5.785; *p* = 0.026; η² = 0.224). However, these effects were qualified by the crucial Test × Modality interaction (F (1, 20) = 6.265; *p* = 0.021; η² = 0.239), illustrated in Figure 4. Pairwise comparisons showed a selective effect of stimulation for high-AQ participants, that is, responses were faster in post-test than in pre-test for false belief stories (t (20) = 4.411, *p* < 0.0001), but not for false photo stories (t (20) = 1.972, *p* = 0.063). As for the low-AQ group, neither the main effects of Test or Modality nor their interaction were statistically significant (all Fs < 1).

### 3.2. Self-Other Distinction Task

In this task, only the reaction time was used as a dependent measure. The high-AQ group showed a nonsignificant trend to respond more slowly than the low-AQ group (M = 3187 ms and M = 2796 ms, respectively; F (1, 40) = 3.33; *p* = 0.075). However, the between-group t-tests for each experimental condition obtained a significant difference: in the first session, high-AQ participants responded more slowly to you-mental questions (M = 3245, SD = 992) than low-AQ participants (M = 2704, SD = 564; t (40) = 2.173, *p* < 0.036). Significant main effects were obtained for Test: F (1, 40) = 21.295; *p* = 0.0001; η² = 0.347 (faster responses in the post-test than in the pre-test), Reference: F (1, 40) = 106.926; *p* = 0.0001 η² = 0.728 (faster responses for you than for other) and Feature: F (1, 40) = 11.553; *p* = 0.002; η² = 0.224 (faster for physical than for mental features). 

However, these main effects were modulated by important three-way interactions. Especially relevant was the Test × Group × Feature interaction: F (1, 40) = 5.063; *p* = 0.030; η² = 0.112, which indicates that high-AQ and low-AQ participants responded differentially in the pre-test and post-test sessions depending on the features of the task. Finally, there was a Group × Feature × Reference interaction: F (1, 40) = 4.828; *p* = 0.034; η² = 0.108. To explore these interactions, new ANOVAs were performed for the high-AQ and the low-AQ groups separately. 

**Figure 5:**
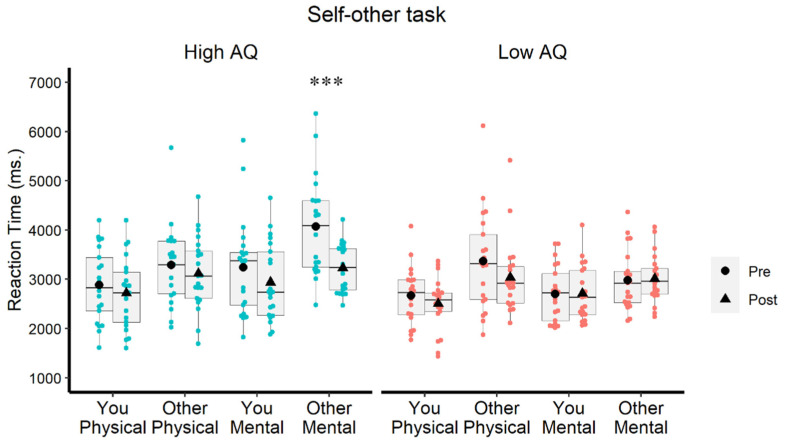
Reaction times in the self-other task in the high-AQ group (**left**) and the low-AQ group (**right**) as a function of Test (pre-post-tDCS), Reference (you, other) and Feature (physical, mental). Only significant effects of test are signaled (*** *p* < 0.0001).

The analysis of repeated measures with the high-AQ group alone showed robust main effects of Test: F (1, 20) = 18.381, *p* < 0.0001; η² = 0.479 (post-test faster than pre-test), Feature: F (1, 20) = 23. 419, *p* < 0.0001, η² = 0.539 (physical faster than mental) and Reference: F (1, 20) = 43.663, *p* < 0.0001, η² = 0.686 (you faster than other). However, a significant Test × Feature interaction was also obtained: F (1, 20) = 4.503; *p* = 0.047, η² = 0.184, indicating that whereas for the mental features, responses were significantly faster in the post-test (M = 3238, SD =655) than in the pre-test (M= 3659, SD = 910; t (20) = 4.239; *p* < 0.0001), for the physical features, the post-test and pre-test (M = 3088, SD = 680; and M = 2919, SD = 666, respectively) did not differ significantly: t (20) = 1.483; *p* = 0.154. This means that the beneficial impact of stimulation was selective over those items that demand the evaluation of mental features. This was corroborated by the fact that the effect of Test in the high-AQ group was constrained to the combination of other-mental: t (20) = 4.239; *p* = 0.0001, while it did not reach significance in the combination of you-mental: t (20) = 1.692; *p* = 0.106. Figure 5 shows the results.

Concerning the analysis with the low-AQ group, a robust main effect of Reference was found: F (1, 20) = 72.050; *p* = 0.0001; η² = 0.783; that is, faster responses were found for you (M = 2646, SD = 554) than for other (M = 3099, SD = 708), although this effect was modulated by an interaction with Feature: F (1, 20) = 4.544; *p* = 0.046; η² = 0.185, since responses to you-physical (M = 2586, SD = 551) were faster than responses to other-physical (M = 3204, SD = 870). Importantly, there was no significant effect of Test (F (1, 20) = 3.648; *p* = 0.071) or its interaction with any other factor, which means that there was no appreciable familiarity or learning effect for low-AQ participants, which serve as controls (Figure 5). 

### 3.3. Correlations

We explored the relationship between self-report measures and performance measures. Specifically, the correlations between the scores in AQ and EQ and the reaction times in the tasks were computed for the pre-test session. This procedure is justified, because at this stage both the high-AQ and low-AQ groups passed exactly the same protocol, before undergoing any treatment with tDCS. As Table 2 shows, the correlations of AQ and EQ scores with the RTs in the mentalizing tasks (false belief, other-mental, you-mental) were significant in the expected direction. That is, the higher the autism score, the slower the responses to mentalizing tasks, and the higher the empathy score, the faster the responses to those same tasks. There was also an unexpected negative correlation between EQ and you-physical judgments.

## 4. Discussion

This study showed for the first time that a multisession excitatory stimulation on the social brain of persons with high levels of autistic traits (high-AQ scores) improves their performance on representative mentalizing tasks. Anodal tDCS was applied over the right temporo-parietal junction, a significant region with ToM functions, in four consecutive sessions, and participants’ performance in false belief and self-other distinction tasks was measured before and after the treatment. High-AQ participants improved after treatment (faster responses) exclusively in those tasks requiring mentalizing; that is, in false belief judgments and you-other distinction of mental features. Interestingly, persons with high AQ did not modify their performance in the tasks with little or no demand for ToM, such as the false photograph or you-other distinction of physical features. This suggests that the beneficial effects of a-tDCS in persons with ASD traits are specifically related to mentalizing processes and cannot be attributed to general learning or familiarity with the tasks. This was corroborated by the fact that in the low-AQ control group that received no active stimulation, there was no improvement in the ToM tasks.

### 4.1. Belief Task

As expected, performance was better in the false photo than in false belief trials; that is, responses were faster and more accurate. This was true for both groups of participants and is consistent with previous results in the literature [9]. It may seem surprising that the high-AQ group and the low-AQ group show similar accuracy on the false belief task. However, the literature on ASD performance in ToM tasks is inconsistent; some studies report poor performance in mentalizing tasks [41,42,43], whereas other studies do not find differences between ASD participants and matched controls in the false belief task [44]. This is especially the case when the task involves a simple situation (like the false belief task) and/or explicit mentalizing instructions [41,45]. Under more naturalistic conditions, such as watching videos of social interactions that require implicit mentalizing inferences, the performance of persons with ASD becomes poorer [44,45]. Despite the simplicity and explicit nature of our false belief task, in the initial session of our study high-AQ participants responded more slowly than low-AQ controls to the false belief task, suggesting that they perform the task correctly at the cost of dedicating more mental resources to it. In the same vein, in the initial session, there was a significant correlation between AQ scores and response time in the false belief task. Importantly, the difference between the high-AQ and low-AQ groups in response speed to false belief stories vanished in the final session after the high-AQ group received active a-tDCS.

The effect of a-tDCS on false belief performance in the high-AQ group is especially relevant here. It suggests that the stimulation of rTPJ has a very selective effect on mentalizing processes and does not affect the false photo task, despite being comparable in logical complexity (dual representations) and inhibitory demands. The critical difference is that, unlike false belief, the false photo task does not require thinking about someone’s thoughts. Nonetheless, the impact of a-tDCS on performance was limited to response times and did not affect the accuracy of the false belief judgments. This could be due to the fact that the false belief task was simple enough to induce a ceiling effect that prevents further improvements with the treatment. In future studies with ASD participants, the use of an implicit false belief task embedded in naturalistic social contexts [44,45] could make it possible for a-tDCS to improve accuracy in mentalizing tasks.

### 4.2. Self-Other Distinction Task

Both participants with high AQ and participants with low AQ scores responded faster to self-referred questions than to other-referred questions. This could indicate the additional cost of mental perspective taking (putting myself in someone else’s shoes) or it could simply be a matter of familiarity (I know myself better than I know anyone else). More important are the differences observed between the two groups of participants. First, the a-tDCS applied to the high-AQ group selectively improved the response speed to questions about mental features referring to another person, while the control group’s performance did not change in any task. That is, stimulating rTPJ in persons with high levels of autistic traits have a selective effect on those questions that are more demanding of ToM inferences. We did not obtain a similar improvement in questions referred to oneself, despite the fact that previous studies have reported that persons with ASD also have impaired self-reference functions [46]. However, consistent with this fact, our correlational data revealed that AQ and EQ scores predict response speed to self-referred mental questions; in other words, higher AQ and lower EQ scores are associated with greater difficulty in mental self-reference. On the other hand, the absence of a-tDCS effects on performance in this task may be due to the specific functional neuroanatomy of self-reference that comprises medial brain structures and, therefore, the stimulation of rTPJ could have a reduced impact [20,21].

### 4.3. Limitations and Future Research Avenues

As stated above, this study is a basic demonstration that a-tDCS applied over rTPJ could improve social cognition in persons with autistic traits. However, as such the study has some limitations that we want to highlight for future research. First, in this study, the high-AQ cut-off score was 30, rather than the standard 32 usually reported in the literature. Second, there are general limitations to non-invasive brain stimulation; for instance, the stimulation is applied to a specific cortical region, whereas potentially relevant subcortical regions cannot be directly stimulated; in addition, the stimulation on the target region overlaps with neighboring regions [47]. These limitations could be partially overcome by using more focal stimulation (rTMS or HD-tDCS) and estimating the information flow by co-registration procedures (TMS-EEG or MEG-EEG). Third, the experimental design could be enriched with a high-AQ control group that would receive sham stimulation between the pre-test and post-test. A high-AQ control group would be more comparable with the group receiving active tDCS and, therefore, the assessment of the stimulation would be more efficient. However, we had a small sample of high-AQ participants available and using a sham stimulation multisession protocol for these persons does not seem ethically appropriate. We chose low-AQ participants as controls here, who were submitted just to the initial and final sessions, both including sham tDCS + tests. This is only a partial control procedure since it would be more appropriate to employ as many sham sessions in the low-AQ group as active sessions in the high-AQ group. In any case, our control group confirms that simple exposure to mentalizing tasks was not responsible for the changes observed in the high-AQ group. Fourth, to fully test the functionality of rTPJ in ToM, it would be useful to apply cathodal tDCS to another group of participants, testing the opposite effects, that is, how inhibition of rTPJ impairs performance in the mentalizing tasks. This would be technically appropriate, to reinforce the causal hypothesis that rTPJ activity governs ToM. However, again it seems ethically unacceptable, especially if the inhibitory stimulation is applied to high-AQ persons over several sessions. Finally, the post-treatment evaluation of the high-AQ group was performed immediately after receiving the last session of tDCS; therefore, we cannot dissociate the momentary effect of the last stimulation from the cumulative effects of the four sessions. In further studies, the duration of the treatment should be extended to at least two or three weeks of daily sessions, except weekends, and the therapeutical evaluation should include a follow-up at least one week after finishing the treatment. Furthermore, it might be useful to collect not only objective performance measures but also more subjective measures of self-reports based on daily life [24].

## 5. Conclusions

Our findings demonstrate the efficacy of tDCS applied to persons with ASD traits, to improve their performance on representative social cognition tasks. We can establish two general consequences of this study. First, it confirms that the rTPJ used as a target for stimulation is functionally involved in the attribution of mental states to others and oneself, which are basic skills of cognitive ToM, necessary to properly navigate the social world. Second, it serves as initial proof that multisession anodal tDCS could be used to improve social cognition in persons with ToM deficits. 

## Figures and Tables

**Table 1:** Examples of false belief and false photograph stories.

Condition	Example
False belief (response “Yes”)	On the morning of the high school party, Sara hid her high-heeled shoes under her dress. Later, while Sara went out shopping, her sister tried on her high-heeled shoes and put them under the bed. When Sara came back, she assumed that her high-heeled shoes were under her dress. Yes No
False belief (response “No”)	The boss ordered a window cleaner to clean the entire building. The cleaner finished the right side, but his platform broke before he could get to the left side. The next morning, the boss came to work. He found out that all the windows were clean. Yes No
False photo (response “Yes”)	Ten years ago, a volcano erupted on a Caribbean island. As a consequence, the lava completely covered the main harbor of the island. Two months ago, a satellite took a picture of the island. In the photo, the harbor is covered by lava. Yes No
False photo (response “No”)	A long time ago, an explorer drew a map of a small island. Since then, the water level has risen and only a small part of the island remains above water. On the explorer’s map, the island seems to be mostly submerged. Yes No

**Table 2:** Correlations between AQ, EQ and reaction times in ToM tasks.

	False Belief	False Photo	Other Mental	Other Physical	You Mental	You Physical
AQ	0.431 **	0.180	0.568 **	0.032	0.360 *	0.218
EQ	−0.414 **	−0.224	−0.567 **	−0.014	−0.416 **	−0.360 *

* *p* < 0.05 ** *p* < 0.01.

## Data Availability

In this link you can check both databases (Self-other Distinction Task and Belief task): https://drive.google.com/drive/folders/1EFkbam8HmJDe1JCIaembb34FAnj6nhcZ?usp=sharing (accessed on 24 December 2021).

## Supplementary Materials

The following supporting information can be downloaded at: https://www.mdpi.com/article/10.3390/brainsci12010030/s1, Figure S1: Reaction times in the belief tasks, in the high-AQ group (left) and the low-AQ group (right) in response Modality and Test (pre- and post-test); Figure S2: Accuracy in the belief tasks, in the high-AQ group (left) and the low-AQ group (right) in response Modality and Test (pre- and post-test); Table S1: Reaction times ANOVA: Group × Test × Response Polarity × Modality; Table S2: Accuracy ANOVA: Group × Test × Response Polarity × Modality
