# Supplementary material for: Multisession Anodal tDCS on the Right Temporo-Parietal Junction Improves Mentalizing Processes in Adults with Autistic Traits
Source: Brain Sci. 2021 Dec 28;12(1):30. doi: 10.3390/brainsci12010030 (PMC8773564; doi:10.3390/brainsci12010030)
Supplement: Supplementary file 1 [file brainsci-12-00030-s001.zip › brainsci-1456974-supplementary.pdf]

Supplementary Material

False belief task including response polarity

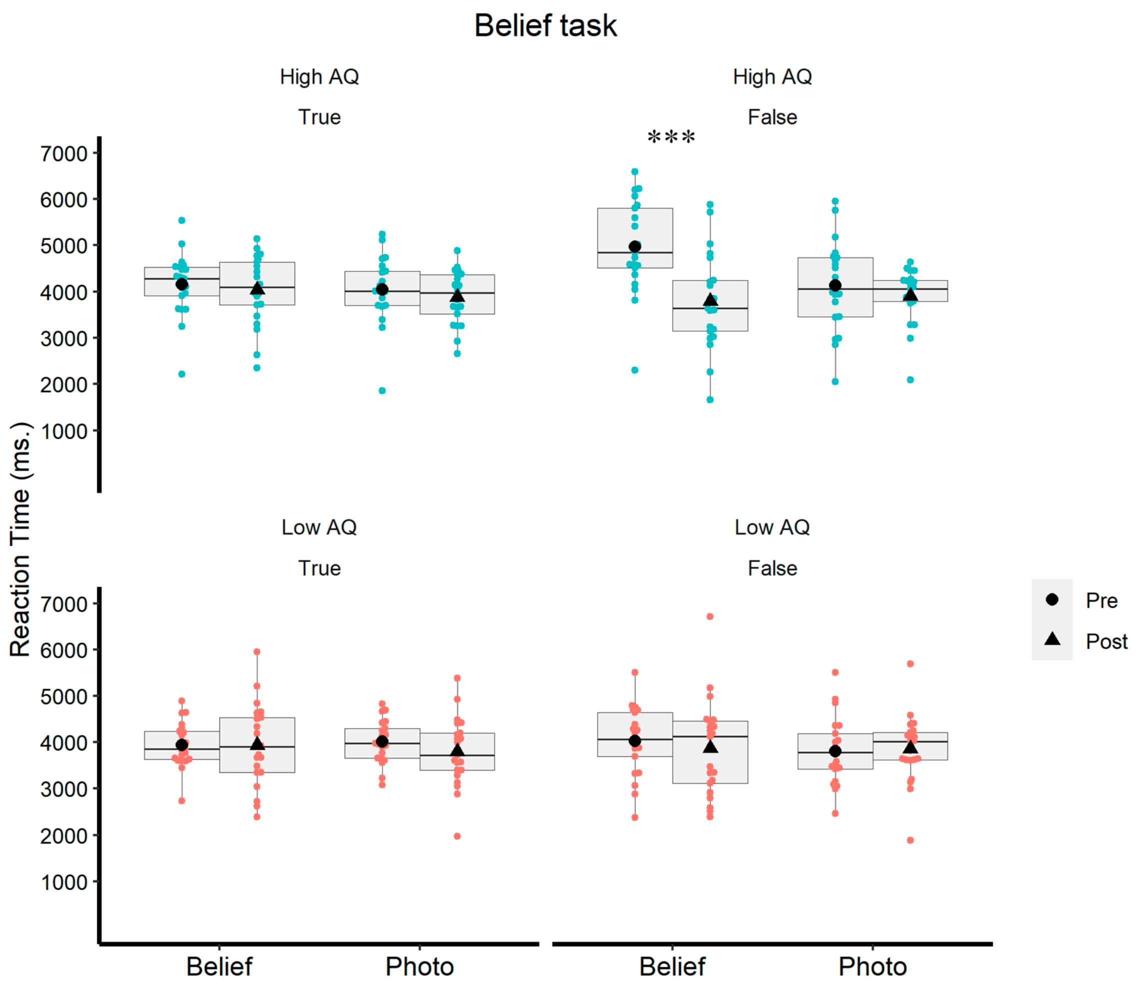

Figure S1. Reaction times in the belief tasks, in the high-AQ group (left) and the low-AQ group (right) in response Modality and Test (pre- and post-test).

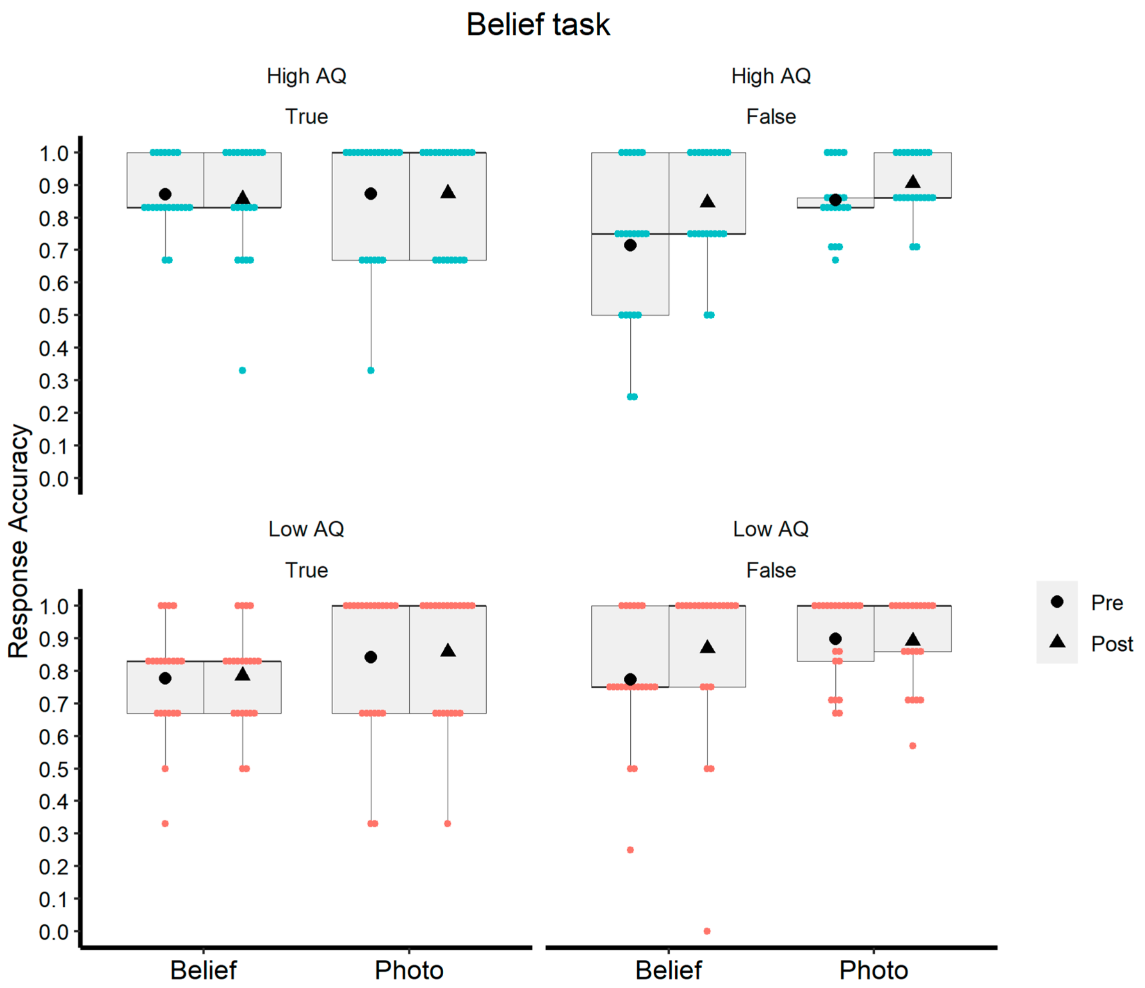

Figure S2. Accuracy in the belief tasks, in the high-AQ group (left) and the low-AQ group (right) in response Modality and Test (pre- and post-test).

Table S1. Reaction times ANOVA: Group x Test x Response Polarity x Modality

SOURCE	SUM OF SQUARES	DF	MEAN SQUARE	F	p	η^2
TEST	5387126.406	1	5387126.406	16.765	.000	.295
TEST * GROUP	2441340.087	1	2441340.087	7.597	.009	.160
Error (TEST)	12853546.539	40	321338.663			
POLARITY	401740.067	1	401740.067	1.093	.302	.027
POLARITY * GROUP	845969.568	1	845969.568	2.302	.137	.054
Error(POLARITY)	14699276.437	40	367481.911			
MODALITY	2228271.702	1	2228271.702	6.092	.018	.132
MODALITY * GROUP	613123.423	1	613123.423	1.676	.203	.040
Error(MODALITY)	14630736.948	40	365768.424			
TEST * POLARITY	1292562.545	1	1292562.545	4.862	.033	.108
TEST * POLARITY * GROUP	1943751.330	1	1943751.330	7.312	.010	.155
Error(TEST * POLARITY)	10633051.052	40	265826.276			
TEST * MODALITY	1100268.509	1	1100268.509	3.014	.090	.070
TEST * MODALITY * GROUP	1007793.198	1	1007793.198	2.760	.104	.065
Error(TEST * MODALITY)	14604383.398	40	365109.585			
POLARITY * MODALITY	477668.809	1	477668.809	1.512	.226	.036
POLARITY * MODALITY * GROUP	116560.838	1	116560.838	.369	.547	.009
Error(POLARITY*MODALITY)	12634453.260	40	315861.331			
TEST * POLARITY * MODALITY	2652885.092	1	2652885.092	6.673	.014	.143
TEST * POLARITY * MODALITY * GROUP	419044.259	1	419044.259	1.054	.311	.026
Error(TEST*POLARITY*MODALITY)	15902391.273	40	397559.782			

Table S2. Accuracy ANOVA: Group x Test x Response Polarity x Modality

SOURCE	SUM OF SQUARES	FD	MEAN SQUARE	F	Sig.	η^2
TEST	.105	1	.105	4.947	.032	.110
TEST * GROUP	.004	1	.004	.199	.658	.005
Error(TEST)	.846	40	.021			
POLARITY	.000	1	.000	.005	.941	.000
POLARITY * GROUP	.140	1	.140	4.893	.033	.109
Error(POLARITY)	1.148	40	.029			
MODALITY	.331	1	.331	17.236	.000	.301
MODALITY * GROUP	.005	1	.005	.282	.598	.007
Error(MODALITY)	.769	40	.019			
TEST * POLARITY	.090	1	.090	1.990	.166	.047
TEST * POLARITY * GROUP	.024	1	.024	.517	.476	.013
Error(TEST*POLARITY)	1.817	40	.045			
TEST * MODALITY	.032	1	.032	1.394	.245	.034
TEST * MODALITY * GROUP	.001	1	.001	.054	.817	.001
Error(TEST*MODALITY)	.924	40	.023			
POLARITY * MODALITY	.046	1	.046	1.367	.249	.033
POLARITY * MODALITY * GROUP	.038	1	.038	1.141	.292	.028
Error(POLARITY*MODALITY)	1.345	40	.034			
TEST * POLARITY * MODALITY	.054	1	.054	3.390	.073	.078
TEST * POLARITY * MODALITY * GROUP	.000	1	.000	.023	.881	.001
Error(TEST*POLARITY*MODALITY)	.640	40	.016			
